# Diversity and distribution of Type VI Secretion System gene clusters in bacterial plasmids

**DOI:** 10.1038/s41598-022-12382-3

**Published:** 2022-05-17

**Authors:** Sergio Morgado, Ana Carolina Vicente

**Affiliations:** Laboratory of Molecular Genetics of Microorganisms, Oswaldo Cruz Institute, Av Brasil 4365 - Manguinhos, Rio de Janeiro, RJ 21040-900 Brazil

**Keywords:** Computational biology and bioinformatics, Genetics, Microbiology

## Abstract

Type VI Secretion System **(**T6SS) is a nanomolecular apparatus that allows the delivery of effector molecules through the cell envelope of a donor bacterium to prokaryotic and/or eukaryotic cells, playing a role in the bacterial competition, virulence, and host interaction. T6SS is patchily distributed in bacterial genomes, suggesting an association with horizontal gene transfer (HGT). In fact, T6SS gene loci are eventually found within genomic islands (GIs), and there are some reports in plasmids and integrative and conjugative elements (ICEs). The impact that T6SS may have on bacteria fitness and the lack of evidence on its spread mechanism led us to question whether plasmids could represent a key mechanism in the spread of T6SS in bacteria. Therefore, we performed an *in-silico* analysis to reveal the association between T6SS and plasmids. T6SS was mined on 30,660 plasmids from NCBI based on the presence of at least six T6SS core proteins. T6SS was identified in 330 plasmids, all belonging to the same type (T6SS^i^), mainly in *Proteobacteria* (328/330), particularly in *Rhizobium* and *Ralstonia*. Interestingly, most genomes carrying T6SS-harboring plasmids did not encode T6SS in their chromosomes, and, in general, chromosomal and plasmid T6SSs did not form separate clades.

## Introduction

Microbial communities are dynamic due to the myriad interactions of their members. In these communities, bacteria can communicate with their surrounding through the Type VI Secretion System. This nanomolecular apparatus has been associated with interbacterial relationships, acting as a toxin (called effectors) delivery vehicle through the cell envelope of a donor bacterium to prokaryotic and/or eukaryotic cells. Several roles have been implicated in this system, such as interbacterial killing and growth inhibition, nutrient scavenging, host colonization, kin discrimination, and acquisition of genetic material^1–4^.

The proteins that assemble the T6SS differ slightly between some species, but generally encompass 13 core conserved proteins (TssA-M), which make up a membrane complex, baseplate, needle spike, and sheath^5^. Bioinformatics analyses of hundreds of bacterial genomes revealed that these conserved components form T6SS gene clusters and are widely distributed among Gram-negative bacteria. In addition, phylogenetic analyses of these T6SS core components showed a clear separation of the T6SS gene clusters from different taxa^6^. Thus, currently, T6SS gene clusters are classified into four T6SS types (T6SS^i-iv^), each with variations in the number of the conserved components. The canonical T6SS^i^, found mainly in *Proteobacteria*, encodes the 13 T6SS core components and is subclassified into six subtypes (i1, i2, i3, i4a, i4b, and i5). T6SS^ii^ and T6SS^iii^ were found exclusively on *Francisella* pathogenicity islands and *Bacteroidetes*, respectively; while T6SS^iv^ was observed in *Amoebophilus*^1,7,8^. Li et al. (2015) showed that this classification scheme can also be achieved by analyzing the TssB protein alone^7^.

The lack of ubiquity, the diversity of T6SSs in the chromosome of a wide variety of genera, and the eventual presence of different types of T6SS in the same bacterial genome suggest that some T6SS clusters are likely to be acquired by horizontal gene transfer. Indeed, the T6SS gene loci are eventually found inside genomic islands^9–11^. Recently, it was shown in *Bacteroidales* that the T6SS presents an extensive intra-ecosystem transfer and multi-species spread due to its association with integrative and conjugative elements^12^. In addition, a few dozens of plasmids were also reported carrying the T6SS^3^.

Therefore, the impact that the T6SS may have on the bacteria fitness led us to question whether plasmids could represent a key mechanism in the spread of the T6SS in bacteria, similarly to what has been inferred for the Type VII Secretion System (T7SS) in *Mycobacteriaceae*^13^. Thus, we performed an *in-silico* analysis to screen the T6SS in all plasmids available from NCBI. We observed a limited distribution of T6SS in the thousands of analyzed plasmids. Most of the T6SS-harboring plasmids were harbored by environmental *Proteobacteria*. Interestingly, most genomes carrying T6SS-harboring plasmids may not encode the T6SS on their chromosomes.

## Results

### Screening of T6SS-harboring plasmids

**Figure 1:**
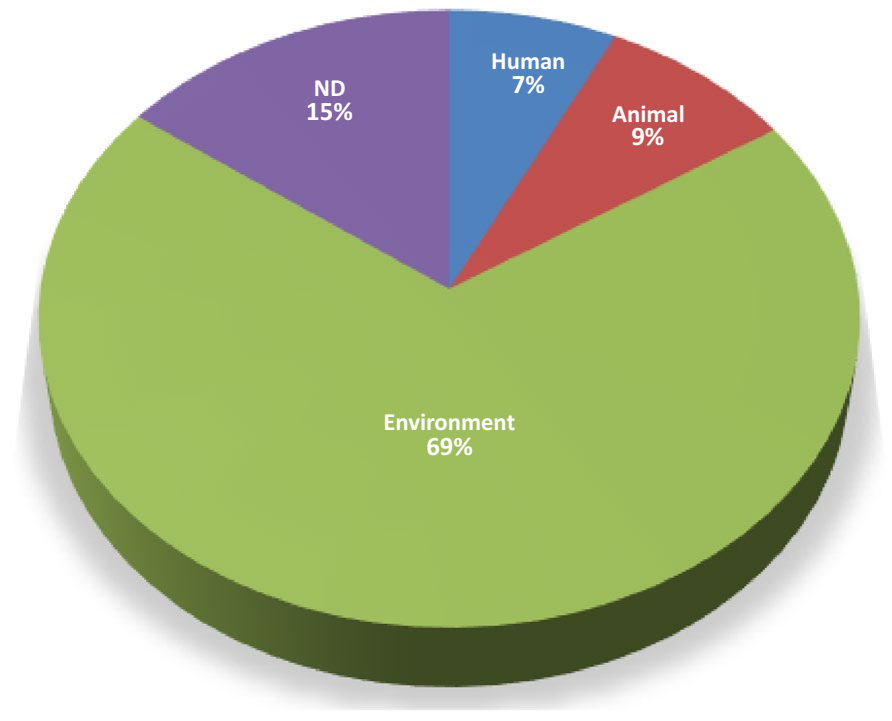
Pie chart of bacterial sources from which sequences were obtained. ND, no data.

We performed an *in-silico* analysis to reveal the association between the T6SS and plasmids. We looked for T6SS gene clusters in 30,660 replicons, classified as plasmids as provided in the NCBI files, based on the presence of at least six of the 11 T6SS core proteins (TssA-M) close to each other. Thus, the T6SS gene clusters, covering regions from ~ 6.2 to ~ 45 kb in size (~ 25 kb median), were identified in 330 plasmids (~ 1% of the dataset) with lengths ranging from 28 kb to 2.7 Mb (907 kb median) and GC content from 26 to 73% (61% median) (Tables 1 and S1). Based on plasmid mobility gene markers (see methods), most of these 330 plasmids were characterized as non-mobilizable (n = 210), while the remainder as conjugative (n = 60) and mobilizable (n = 60). These 330 T6SS-harboring plasmids were in 307 genomes, of which 23 contained two T6SS-harboring plasmids, and encompassed 22 bacterial families from three phyla, *Acidobacteria* (1/330), *Gemmatimonadetes* (1/330), and *Proteobacteria* (328/330) (Tables 1 and S1). Within the phylum *Proteobacteria*, the T6SS-harboring plasmids were prevalent in α-*Proteobacteria* (n = 105), β-*Proteobacteria* (n = 127), and γ-*Proteobacteria* (n = 80), while less prevalent in ε-*Proteobacteria* (n = 16) (Table 1). As the plasmids had a wide range of sizes, from Kb to Mb, we analyzed these groups separately (Table 2). In addition to the differences in median size and GC content, the Kb size group had proportionally more mobilized and conjugative plasmids. Moreover, the Mb size group was concentrated in only five bacterial families, with a prevalence of *Burkholderiaceae* (74%), while the other group was distributed in 20 families, with a prevalence of *Enterobacteriaceae* (29%) and *Rhizobiaceae* (25%). Among the species with the highest number of T6SS-harboring plasmids, only five species had more than 10 plasmids, of which *Ralstonia solanacearum* had the highest absolute (n = 102) and relative abundances (~ 80%) (Table S2). In addition, some species that have thousands of sequenced plasmids had a low prevalence of T6SS gene clusters in these elements, such as *Escherichia coli* (28/4709 plasmids) and *Klebsiella pneumoniae* (3/3431 plasmids) (Table S2). Most genomes carrying T6SS-harboring plasmids are from bacteria that have been isolated from the environment, including roots, soils, water, seeds, plants, and foods, while few have been isolated from humans or animals (Fig. 1 and Table S1). We also investigated the presence of the T6SS in the chromosome of the 307 genomes that carried T6SS-harboring plasmids to verify whether plasmid T6SS was unique in the bacterial genome, and we observed the presence of T6SS (gene clusters with at least six T6SS core proteins) on 70 chromosomes (~ 22%).

**Table 1:** Features of T6SS-carrying plasmids.

Families	Class	Number of plasmids	Median size (kb)	Median GC	Prevalent T6SS type
*Acidobacteriaceae*	Acidobacteriia	1	475	0.6	i4b
*Aurantimonadaceae*	α-Proteobacteria	1	488	0.68	i5
*Azospirillaceae*	α-Proteobacteria	10	1,751	0.68	i4a
*Burkholderiaceae*	β-Proteobacteria	126	2,017	0.67	i4b
*Campylobacteraceae*	ε- Proteobacteria	16	122	0.26	i1
*Chromatiaceae*	γ-Proteobacteria	1	484	0.66	i3
*Enterobacteriaceae*	γ-Proteobacteria	50	141	0.47	i2
*Erwiniaceae*	γ-Proteobacteria	4	326	0.52	i2
*Gemmatimonadaceae*	Gemmatimonadetes	1	1,106	0.73	i4b
*Halomonadaceae*	γ-Proteobacteria	1	1,833	0.55	i1
*Moraxellaceae*	γ-Proteobacteria	1	127	0.41	i3
*Phyllobacteriaceae*	α-Proteobacteria	6	542	0.6	i5
*Pseudoalteromonadaceae*	γ-Proteobacteria	3	899	0.41	i5
*Pseudomonadaceae*	γ-Proteobacteria	1	371	0.55	i1
*Rhizobiaceae*	α-Proteobacteria	69	655	0.59	i3
*Rhodobacteraceae*	α-Proteobacteria	12	222	0.67	i3
*Rhodocyclaceae*	β-Proteobacteria	1	28	0.60	i4b
*Rhodospirillaceae*	α-Proteobacteria	1	692	0.68	i5
*Roseobacteraceae*	α-Proteobacteria	5	148	0.62	i3
*Thalassospiraceae*	α-Proteobacteria	1	908	0.54	i1
*Vibrionaceae*	γ-Proteobacteria	10	1,504	0.45	i1
*Yersiniaceae*	γ-Proteobacteria	9	553	0.52	i4b

**Table 2:** Differences of Mb and Kb plasmid groups.

Features	Mb plasmids	Kb plasmids
# Plasmids	158	172
Median size	1.99 Mb	323 Kb
Median GC%	0.67	0.57
# Conjugative	20	40
# Mobilizable	17	43
# Non-mobilizable	121	89
# Families	5	20
Prevalent families	*Burkholderiaceae* (n = 118)	*Enterobacteriaceae* (n = 50)
		*Rhizobiaceae* (n = 44)
# Plasmids with rRNA	95	7
# Plasmids with tRNA	141	38
# Plasmids with metabolite clusters	100	30

As the T6SS gene clusters are eventually found in GIs, we screened the 330 T6SS-harboring plasmids for the presence of GIs. The detection method considered the dinucleotide composition and the presence of mobility genes (integrase, transposase, resolvase, and recombinase) in the regions. In total, 274/330 plasmids were predicted to contain GIs. However, only GIs from three plasmids (NC_008378.1, NC_013855.1, and NZ_AP023206.1) encompassed the T6SS gene clusters.

### Plasmid T6SS classification

**Figure 2:**
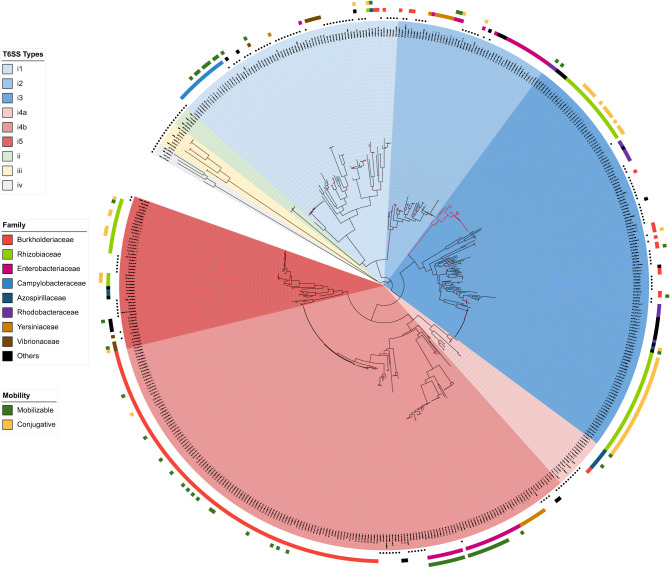
Phylogenetic relationship of plasmid T6SS based on TssB core component protein and maximum likelihood method. The sequences are divided into four types (T6SS^i-iv^) and six subtypes (i1, i2, i3, i4a, i4b, and i5) by colored backgrounds. The chromosomal reference sequences have black circles adjacent to their labels. The red branch represents sequences assigned to the i3 subtype, but which are not grouped with the i3 reference sequences. The colored blocks closest to the tree represent the bacterial families from which the sequences were obtained. The predicted mobility of the plasmids harboring the T6SS gene clusters is indicated by the outer colored blocks. Bootstrap values above 70 are shown as red circles in the middle of the branches.

The T6SS classification scheme, based on the sequence of the TssB component (VipA or IglA), showed that all the T6SS harbored by the 330 plasmids belonged to the T6SS^i^, with an abundance of i4b and i3 subtypes. A maximum-likelihood tree, based on the TssB sequences of these plasmids and chromosomal references, showed the clustering of the different T6SS^i^ subtypes, each with related groups of taxa (Fig. 2). For most taxa, there was no association with a unique T6SS^i^ subtype, since different subtypes were identified in the same taxon, such as *Rhizobium* (i1, i3, and i5), *Rahnella* (i1, i2, and i4b), *Paraburkholderia* (i2, i3, i4a, and i4b), and *Azospirillum* (i1, i4a, and i5). Only the *Campylobacteraceae* plasmids were associated with only one T6SS^i^ subtype (i1). Interestingly, plasmid T6SS from this bacterial family seems conserved, as seen by the tree branches, even for strains isolated from different sources, countries, and years (Table S1). Curiously, the two non-*Proteobacteria* sequences (*Acidobacteria* and *Gemmatimonadetes*) clustered in the same clade in a branch of the i4b subtype. Most of the defined clusters, based on the TssB protein, presented chromosomal and plasmid sequences, suggesting some interplay of the T6SS of these types of replicons. However, few clusters contain only plasmid or chromosome sequences. Particularly, there is a cluster with sequences from different families (*Rhodobacteraceae*, *Phyllobacteriaceae*, *Rhizobiaceae*), plasmid sizes and classified as subtype i3 that was positioned apart from other sequences of the i3 subtype (Fig. 2, red branch), which could represent a new T6SS subtype, until now, plasmid-exclusive T6SS subtype.

**Figure 3:**
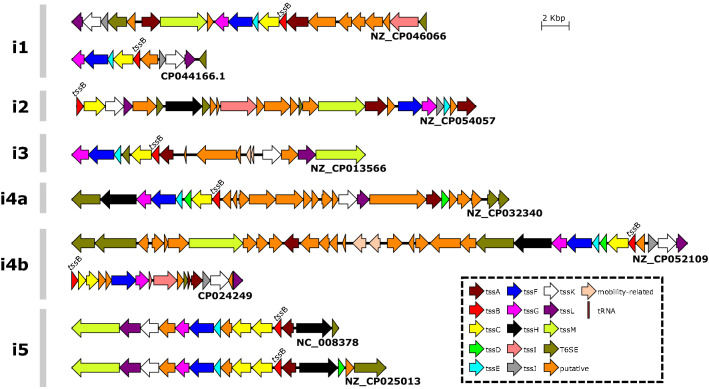
Genetic architecture of T6SS gene loci of different type i subtypes.

The genetic organization of the T6SS loci is quite variable in size, number of genes, and orientation, even considering the same subtype. In addition, at some loci, it is possible to observe that there are duplications of some core genes, such as *tss*A and *tss*C (Fig. 3). This shows that the regions of these T6SSs have gene plasticity.

### Gene content of T6SS-harboring plasmids

As the T6SS provides fitness and colonization advantages, we also searched for other plasmid cargo genes, such as T6SS effectors (T6SEs), virulence and antibiotic resistance genes, and secondary metabolites gene clusters. Regarding the presence and type of T6SS effectors, of the 330 plasmids, 262 encoded 114 types of T6SEs (based on SecReT6 database IDs), ranging from one to 30 effectors per plasmid (median of 12 T6SEs). According to the SecReT6 database, most of these 114 types of T6SEs (n = 88) have no assigned function, while the others were related to periplasmatic, cytoplasmatic, and environmental effectors: six were associated with amidase, six with DNase and RNase, five with lipase, one with glycoside hydrolase, four with metal ion acquisition, and three with peptidase (Table S3). Lipase effectors were widely present in *Ralstonia solanacearum* plasmids (79/102).

The search for virulence and antibiotic resistance genes in these plasmids revealed that 12/330 and 31/330 encoded genes associated with antibiotic resistance and virulence (disregarding the T6SS genes), respectively. Plasmids with the most antibiotic resistance and virulence genes were from bacteria recovered from humans, animals, and food (Tables S4 and S5). Interestingly, it was identified in 132 plasmids, gene clusters with 100% similarity to 13 metabolite types of non-ribosomal peptide synthetase (NRPS), ectoine, terpene, etc. (Table S1). These metabolites were associated with siderophores, osmotic protection, photosynthesis, antimicrobial and antifungal activities; and each type of metabolite gene cluster was only found on plasmids of a specific taxon, e.g., ralsolamycin and rhizoxin in *Ralstonia*, vicibactin in *Rhizobium*, and carotenoid in *Pantoea* (Table S1). Curiously, hundreds of these plasmids, of at least nine bacterial families, had genes associated with protein syntheses, such as rRNA (n = 102) and tRNA (n = 179). These genes were more associated with Mb-sized plasmids (Table 2).

## Discussion

The T6SS is a key apparatus in inter-microbial interactions to compete for niches, being generally encoded by dozens of genes, which can vary depending on the taxon. To date, the T6SS has been identified in several genera of seven phyla of Gram-negative bacteria, *Acidobacteria*, *Bacteroidetes*, *Deferribacteres, Gemmatimonadetes*, *Nitrospirae*, *Planctomycetes,* and *Proteobacteria*, being abundant in the latter^1,7,14–16^. Although widely present and diverse in these bacterial phyla, the T6SS has a patchy distribution, not being ubiquitous in all these bacterial genomes^8,14,17,18^. This suggests an association of the T6SS with horizontal gene transfer. Indeed, the T6SS gene clusters are eventually located in genomic islands, which have the potential to be transferred, as a unit, to other cells^9^. Thus, other genetic elements could act as carriers of the T6SS. In fact, some T6SS from *Bacteroidales* are associated with ICEs^12^, and so far, twenty-nine plasmids with T6SS have been reported^3^, some of them functionally tested^19–21^.

Here, to determine the distribution of T6SS in all bacterial plasmids, we mined thousands of plasmids available in NCBI and identified hundreds (n = 330) of T6SS-harboring plasmids, mainly in *Proteobacteria*. The T6SS was present in plasmids with a wide range of sizes (27 kb–2.8 Mb), which could imply different fitness costs (depending on the size of the plasmid), thus imposing a restriction on the vertical and horizontal replication of these plasmids. Furthermore, for bacteria that carry smaller plasmids with T6SS, they would be more likely to show a T6SS-associated phenotype due to the higher copy number of this gene set. Although present in several phyla and genera^14^, the T6SS distribution in plasmids is limited, as only ~ 1% of them encoded this secretion system. Some factors may contribute to this phenomenon: (i) the dissemination of the T6SS via plasmids, at least in *Proteobacteria*, seems to have barriers, since bacteria with chromosomal T6SS (abundant in *Proteobacteria*) may present a defense mechanism via T6SS against the acquisition of new plasmids^3^; (ii) carrying an extra copy of the T6SS does not seem advantageous if the bacterium already has a chromosomal copy, as it is a niche-specific system and different T6SSs do not confer different functions, depending more on the effectors that are secreted (unless these T6SSs are regulated differently)^10^. Furthermore, most of the T6SS-harboring plasmids identified in this study are large (> 100 kb), which would likely pose barriers to their acquisition and maintenance.

Previously, Abby et al. (2016) showed that the T6SS was more prevalent in γ-*Proteobacteria* than in α- and β-*Proteobacteria* (genomes with and without plasmids)^14^, and curiously, here, we observed that the plasmid T6SS prevails in α- and β-*Proteobacteria*. This difference is probably due to the datasets used, since Abby et al. (2016) used only complete genomes (some with associated plasmids)^14^, and we considered only plasmids. On the other hand, plasmids with T6SS were more prevalent in γ-*Proteobacteria* when considering those carrying less than nine T6SS genes, indicating that a degradation process may be underway. Here we also observed the T6SS in two plasmids from other phyla, *Acidobacteria* and *Gemmatimonadetes*, which could indicate their acquisition from a phylum in which plasmids carrying T6SS are prevalent. Interestingly, most T6SS-harboring plasmids (~ 78%) were present in bacterial genomes that did not encode chromosomal T6SS, indicating that a mobile platform (plasmids in this case) may represent the sole source of the T6SS for some bacterial genomes.

Of the four T6SS types (T6SS^i-iv^), T6SS^i^ was the only one found in the plasmids. Indeed, this type is also the most common type of T6SS in *Proteobacteria*^14^. Considering T6SS^i^ subtypes, i1 and i2 subtypes prevail in the chromosomes and plasmids of Abby et al. (2016) dataset^14^, while the i4b and i3 subtypes prevail in the plasmids, the first (i4b) being predominant in a specific taxon, *Ralstonia solanacearum* (Table S1). Again, this difference must be related to the datasets used, as our dataset contained more α-*Proteobacteria* and β-*Proteobacteria*, while Abby et al. (2016) had a dataset with a prevalence of γ-*Proteobacteria*. Interestingly, despite the T6SS^i^ being the canonical one, which would encode the 13 core components of T6SS, here we observe a wide variability of this number in the plasmids with the T6SS^i^. Of note, there was a subcluster in the i3 subtype clade with dozens of plasmid sequences that were not closely related to any reference chromosomal sequence (e.g., NZ_CP025431.1, NZ_CP070369.1, NZ_ CP015063.1, NC_014918.1, NZ_CP006880.1, NZ_CP013589.1). Most of these sequences belonged to environmental bacteria and could be evolving independently of the others i3 subtype sequences.

Considering the gene cargo of the analyzed plasmids, we did not observe in most of them a prevalence of resistance or virulence genes (disregarding the T6SS). The few plasmids identified carrying resistance and virulence genes were mainly from bacteria isolated from human or animal hosts. Indeed, clinical T6SS-positive bacteria were observed to have a higher resistance and frequency of virulence genes^17^. Among the T6SS effectors identified in the plasmids, most of them play a role in virulence, but also in bacterial competition under stress conditions, such as ModA, which provides a growth advantage under anaerobiosis^22^, and TseZ, which is a zinc-scavenging protein under oxidative stress conditions^23^. Thus, unless these T6SSs play a virulence role in their host niche, these plasmids would be more related to ecological roles. Even because some of them also encode secondary metabolites related to survival and protection. The ecological gene cargos of the T6SS-harboring plasmids identified here contrast with virulent T6SS-harboring plasmids from clinical bacteria, such as *Cronobacter* spp. and *Campylobacter jejuni*^24,25^.

Although 120/330 of the T6SS-carrying plasmids were predicted to be conjugative or mobilizable, their median size (~ 435 kb) would represent a natural restriction on transmission. Thus, the mobility marker genes of these plasmids could be associated with other mobile elements, such as genomic islands. In fact, GIs have been predicted on hundreds of T6SS-harboring plasmids. However, only three GIs encompassed the T6SS gene clusters, suggesting that mobilization of the T6SS to plasmids may have taken place a long time ago and there are no more traces (based on the method used for detection), or there are other mechanisms of mobilization of the T6SS to plasmids.

Finally, in dozens of these T6SS-harboring plasmids we identified genes mainly associated with chromosomes, such as rRNA, and this, added to the fact that most of them are megabases in size (~ 60% of the Mb size group had rRNA genes vs ~ 4% of the Kb size group), raised the question of whether they were in fact plasmids or another type of replicon. Recently, Schmartz et al. (2022)^26^ analyzed putative plasmids in terms of the presence of ribosomal genes to identify mislabelled sequences. Thus, some sequences initially considered in our study were filtered. Even so, some sequences containing ribosomal genes remained in our analysis, as they have other elements that characterize them as plasmids (for example, the presence of the *rep* gene). In fact, some of the genera identified here were associated with secondary essential replicons (secondary chromosomes and chromids), such as *Burkholderia*, *Cupriavidus*, *Ensifer*/*Sinorhizobium*, *Pantoea*, *Ralstonia*, *Rhizobium*, *Vibrio*^27,28^. It can be speculated that this could explain the large number of elements with T6SS predicted as non-mobilizable, since chromids, for example, tend to lose the ability to transmit horizontally, thus becoming "stuck" to a particular genome. In fact, non-mobilizable megaplasmids can undergo processes to become chromids^28^. Thus, the identity of these replicons of these organisms is still under debate (megaplasmid, chromid, or secondary chromosome).

Therefore, our findings do not fully support the hypothesis that T6SS spread within bacteria was plasmid-mediated, as occurred with T7SS in *Mycobacteriaceae*^13^. Furthermore, most T6SSs from the chromosomal and plasmid compartments do not seem to evolve independently, as observed in the phylogeny, reinforcing that the T6SS regions may be under constant gene flow. Even so, the evidence gathered here points to the involvement of mobile platforms in the spread of the T6SS within bacteria.

## Methods

### Plasmid dataset

A total of 30,660 replicons, classified as plasmids, were obtained from the NCBI Refseq database (https://www.ncbi.nlm.nih.gov/genome/browse/#!/plasmids/) in Sep-2021, and encompassed more than 20 bacterial phyla (Table S6). Since some NCBI sequences tagged as plasmids are mislabeled chromosomal sequences, we removed from our dataset those sequences that were not present in the PLSDB^26^, a curated database of bacterial plasmids fed from the NCBI nucleotide database. Some replicons of some genera presented sizes in megabases (e.g., *Ralstonia solanacearum* strain RS10 plasmid unnamed with ~ 2 Mb; *Rhizobium phaseoli* strain BS3 plasmid pBS3d with ~ 1.1 Mb). The identity of these replicons of these organisms is still under debate (megaplasmid, chromid, or secondary chromosome)^28^, but as they were assigned as plasmids by the authors and they are present in a curated plasmid database (PLSDB), we considered them for the analysis.

### T6SS identification, classification, and phylogeny

The 30,660 plasmids were annotated using Prokka v1.12^29^ to predict their proteomes, which were screened for the T6SS core proteins. This step was performed using the hmmsearch program^30^ considering an e-value of 1e-10. In total, hmm profiles of 11 Clusters of Orthologous Groups of proteins (COGs) comprehending the T6SS core genes were used and are listed in Table 3. We considered 11 COGs instead of 13, as COG3501 (VgrG-TssI) and COG0542 (ClpV-TssH) were shown not to be T6SS specific^31^. Plasmids that encoded at least six of 11 T6SS core proteins close to each other were considered carriers of T6SS gene clusters. We considered six genes as a cut-off value because in previous analyses we observed that clusters with less than six T6SS core genes generally did not have *tss*B and/or *tss*C, which are well conserved and used in the classification of T6SS, which would suggest that these smaller clusters could be degraded and non-functional. In addition, these parameters were also used by Li et al. (2015).

**Table 3:** List of T6SS core genes used.

Protein	COG	Synonym	Domain access
TssA	COG3515	impA/vasJ	PF06812
TssB	COG3516	impB, vipA	PF05591
TssC	COG3517	impC, vipB	TIGR03355.1
TssD	COG3157	Hcp	PF05638
TssE	COG3518	mpF, vasS	PF04965
TssF	COG3519	impG, vasA	PF05947
TssG	COG3520	impH, vasB	PF06996
TssJ	COG3521	vasD, lip	PF12790
TssK	COG3522	impJ, vasE	PF05936
TssL	COG3455	ompA/dotU	PF09850
TssM	COG3523	vasK, icmF	PF06744

The classification of plasmid-borne T6SSs was based on the TssB protein since it was observed that this protein alone may be a suitable classification marker. For each plasmid T6SS, its TssB sequence was extracted and submitted to SecReT6 web platform (https://bioinfo-mml.sjtu.edu.cn/SecReT6/phylogenetic_analysis.php) in the T6SS classification tool^7^. Furthermore, the type of T6SS that these TssB sequences would represent could be observed in a phylogeny of TssB along with reference sequences of known T6SS types.

The TssB phylogeny encompassed all TssB sequences identified in the plasmids along with 152 chromosomal reference sequences. Initially, the TssB sequences were aligned by MAFFT v7.453^32^, and the low-quality alignment columns were removed using GUIDANCE2 v2.02^33^. Next, the TssB alignment was submitted to IQTree v1.6.12^34^ to obtain a maximum likelihood tree, which used the best-fit amino acid substitution model (WAG + G4) and 1000 ultrafast bootstrap replicates^35^. The tree was visualized using the iTOL web platform (https://itol.embl.de)^36^.

For all software used in this study, the default parameters were applied, except when e-value, coverage, or identity was mentioned.

### Characterization of T6SS-carrying plasmids

T6SS-positive plasmids were characterized concerning their gene cargo: clusters of secondary metabolites were mined using antiSMASH v6^37^; virulence and antibiotic resistance genes were screened by ABRicate (https://github.com/tseemann/abricate) based on VFDB^38^ and CARD^39^ databases (Sep-2021); The T6SS effectors, consisting of 294 experimentally verified T6SEs from the integrated database SecReT6^7^ (Sep-2021), were searched considering the whole plasmid sequences using BLASTP with 50% identity and 60% coverage. Genomic islands and ICEs were surveyed in the plasmids using IslandPath-DIMOB v1.0.0^40^ and ICEfinder web-based tool^41^, respectively.

The plasmids also had their mobility predicted based on the presence of gene markers, such as relaxase and Type IV Secretion System (T4SS)-like genes (e.g., VirB4 and VirD4), as described^42^. Proteins that encoded these genes were surveyed with hmm profiles using the hmmsearch program^30^ considering an e-value of 1e-10. The hmm profiles encompassed relaxases (PF03389, PF05713, PF01076, PF03432, PF08751, PF07514) and T4SS-like genes (PF12846, PF02534, PF12615, PF12642, PF12696, PF10412) of different conserved domains^43–45^. Plasmids that encoded a relaxase gene and that did not encode VirB4 and/or VirD4 were considered mobilizable, while those encoding relaxase, VirB4, and VirD4 were considered conjugative. Plasmids lacking relaxases were considered incapable of self-mobilization, non-mobilizable^42,44^. However, it is possible that unknown origin of transfer (oriT) sequences are present in these plasmids, which would allow their mobilization only in a relaxase-*in trans* mechanism^46,47^.

## Supplementary Information

Supplementary Information.

## Data Availability

The dataset analyzed during the current study is available on the NCBI plasmid database (https://www.ncbi.nlm.nih.gov/genome/browse/#!/plasmids/) and is listed in Table S6.
